# Shotgun Proteomics of Ascidians Tunic Gives New Insights on Host–Microbe Interactions by Revealing Diverse Antimicrobial Peptides

**DOI:** 10.3390/md18070362

**Published:** 2020-07-13

**Authors:** Ana Matos, Dany Domínguez-Pérez, Daniela Almeida, Guillermin Agüero-Chapin, Alexandre Campos, Hugo Osório, Vitor Vasconcelos, Agostinho Antunes

**Affiliations:** 1 CIIMAR/CIMAR, Interdisciplinary Centre of Marine and Environmental Research, University of Porto, Terminal de Cruzeiros do Porto de Leixões, Av. General Norton de Matos, s/n, 4450-208 Porto, Portugal; anabastosmatos@gmail.com (A.M.); dany.perez@ciimar.up.pt (D.D.-P.); danielaalmeida23@gmail.com (D.A.); gchapin@ciimar.up.pt (G.A.-C.); amoclclix@gmail.com (A.C.); vmvascon@fc.up.pt (V.V.); 2 Biology Department, Faculty of Sciences, University of Porto, Rua do Campo Alegre, s/n, 4169-007 Porto, Portugal; 3 i3S-Instituto de Investigação e Inovação em Saúde, Universidade do Porto, Rua Alfredo Allen, 208, 4200-135 Porto, Portugal; hosorio@ipatimup.pt; 4 Ipatimup, Institute of Molecular Pathology and Immunology of the University of Porto, Rua Júlio Amaral de Carvalho, 45, 4200-135 Porto, Portugal; 5 Department of Pathology and Oncology, Faculty of Medicine, University of Porto, Al. Prof. Hernâni Monteiro, 4200-319 Porto, Portugal

**Keywords:** ascidians, shotgun proteomics, Q-Exactive, Orbitrap, Proteome Discoverer, MaxQuant, AMPs, biotechnological potential

## Abstract

Ascidians are marine invertebrates associated with diverse microbial communities, embedded in their tunic, conferring special ecological and biotechnological relevance to these model organisms used in evolutionary and developmental studies. Next-generation sequencing tools have increased the knowledge of ascidians’ associated organisms and their products, but proteomic studies are still scarce. Hence, we explored the tunic of three ascidian species using a shotgun proteomics approach. Proteins extracted from the tunic of *Ciona* sp., *Molgula* sp., and *Microcosmus* sp. were processed using a nano LC-MS/MS system (Ultimate 3000 liquid chromatography system coupled to a Q-Exactive Hybrid Quadrupole-Orbitrap mass spectrometer). Raw data was searched against UniProtKB – the Universal Protein Resource Knowledgebase (Bacteria and Metazoa section) using Proteome Discoverer software. The resulting proteins were merged with a non-redundant Antimicrobial Peptides (AMPs) database and analysed with MaxQuant freeware. Overall, 337 metazoan and 106 bacterial proteins were identified being mainly involved in basal metabolism, cytoskeletal and catalytic functions. 37 AMPs were identified, most of them attributed to eukaryotic origin apart from bacteriocins. These results and the presence of “Biosynthesis of antibiotics” as one of the most highlighted pathways revealed the tunic as a very active tissue in terms of bioactive compounds production, giving insights on the interactions between host and associated organisms. Although the present work constitutes an exploratory study, the approach employed revealed high potential for high-throughput characterization and biodiscovery of the ascidians’ tunic and its microbiome.

## 1. Introduction

In the last years, the knowledge about marine resources and their associated ecological and biotechnological potential has been increasing [1,2,3,4,5,6]. Tunicates are one of the marine invertebrate groups that have been contributing to scientific advance in the biotechnology field. Tunicata, subphylum of Chordata (along with cephalochordates and vertebrates), are divided into three main classes: Ascidiacea, Thaliacea, and Appendicularia [7]. The close proximity to vertebrates is detected in the larval phase of solitary ascidians with the presence of pharyngeal gill slits, notochord and dorsal nerve cord, some phenotypic characteristics associated with vertebrates [8,9].

The abovementioned characteristics explain the interest and curiosity by the scientific community in those organisms. Ascidians, solitary and colonial forms, are sessile filter-feeders widely dispersed in marine environments with an invasive potential associated to their biofouling activity [10,11]. These organisms are surrounded by an outer tunic mainly composed of a cellulose matrix with diverse free cells distributed within it [12].

Ascidians tunic microbiome has been the subject of intense research in the last years due to its biotechnological potential [13,14,15,16,17]. In fact, several compounds have been isolated and their bioactivity analyzed. Noteworthy are the alkaloid trabectedin and dehyrodidemnin B, plitidepsin, initially isolated from *Ecteinascida turbinata* and from *Aplidium albicans*, respectively; both applied in cancer treatment [18]. The isolated ascidians compounds are suggested to be from microorganisms source being produced as defense against predators and/or against other organisms [19]. Furthermore, in the literature, some of the reported interactions for those microorganisms’ associations are usually the nourishment and protection capacity; assured by the host, while the associated microorganisms may contribute, among other functions, to nitrogen cycle and defense against ultraviolet radiation [15,20]. To date, only a few obligate symbioses occurring in ascidians have been reported: *Prochloron* spp. with several members of Didemnidae family and *Candidatus* Endolissoclinum faulkneri with the colonial ascidian *Lissoclinum patella* [21,22,23,24].

Nowadays, next-generation sequencing techniques and metagenomics approaches are becoming popular allowing also the analysis of uncultivable species widening the spectral of the studied organisms [25,26,27]. The application of transcriptomics and proteomics approaches in ascidians microbiome studies is still scarce [28]. Transcriptomes from model ascidians such as *Ciona intestinalis* and *Botryllus schlosseri* have been studied to understand several developmental and evolutionary aspects in chordates. The transcriptome of *B. schlosseri* at different levels of blastogenetic cycle has been analyzed to understand this asexual developmental strategy [29,30,31]. The transcriptome and proteome of either premature and mature ovaries, and embryonic stages of *C. intestinalis* have been reported [32,33,34]. The study of transcriptomes has been applied to understand the effects of several environmental factors in *C. intestinalis* ovaries [35]. Despite not being so frequent, transcriptomics and metabolomics approaches have been applied to study regulatory pathways of specific associated organisms as *Prochloron* and *Acaryochloris* in the surface and underside of the host *Lissoclinum patella* [28]. Proteogenetics also allowed the discovery of new cyanobactin pathways, as well as the association of the relationship between phylogenies of hosts and *Prochloron* [22]. Conventionally, integrated liquid chromatography-tandem mass spectrometry (LC-MS), complementing proteomics and transcriptomics has been the most applied approach to analyze the chemical diversity of metabolites in ascidians [36]. Recently, shotgun proteomics approach was applied to test the stress response of two solitary ascidians in different environmental conditions, identifying potential proteins to be developed as biomarkers of stress response [37].

Due to the high potential within proteomics approaches and to overcome the scarce number of those studies to characterize ascidians’ microbiome, the present work aims to apply a shotgun proteomics approach to profile the proteome present in the tunic of three ascidians’ species, also allowing the identification of antimicrobial peptides from bacteria. The outcomes revealed the main metabolic pathways give clues of potential interactions occurring between associated microorganisms and hosts.

## 2. Results

### 2.1. Protein Identification

**Figure 1:**
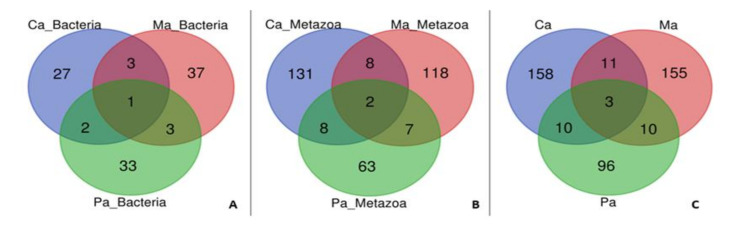
Venn diagrams showing unique and shared proteins among the three ascidians’ species (Ca—*Ciona* sp., Ma—*Molgula* sp., Pa—*Microcosmus* sp.) identified with Proteome Discoverer 2.2.0.388 software (Thermo Scientific) and searched against the Universal Protein Resource Knowledgebase (UniProtKB) for the Metazoa and Bacteria taxonomic selection (2018_07 release). (**A**) Bacteria section; (**B**) Metazoa section; (**C**) Total of proteins retrieved from both Bacteria and Metazoa sections.

Through shotgun proteomics analysis, the proteins present in three ascidians tunic samples were identified using the Proteome Discoverer software (Supplementary Dataset S1). Altogether, 443 unique proteins were identified, of which 337 proteins were retrieved from metazoan section, while 106 corresponded to bacterial proteins (Figure 1). In *Ciona* sp., a total of 182 proteins were identified, of these 33 were bacterial and 149 were metazoan proteins. In *Molgula* sp., 44 bacterial and 135 metazoan proteins were detected, giving a total of 179 proteins, while in *Microcosmus* sp., 119 proteins were identified, 39 of these were bacterial and 80 were metazoan proteins. A total of 34 proteins was shared among the three ascidians’ specimens, 25 metazoan and 9 bacterial proteins (Figure 1, Table 1). However, only 3 of them (1 bacterial and 2 metazoan proteins) were shared by all the studied species. Most of these proteins are part of a cytoskeleton involved in DNA packaging and/or in basal metabolism (Table 1).

### 2.2. Gene Ontology (GO) Annotation of Proteins Identified with Proteome Discoverer

**Figure 2:**
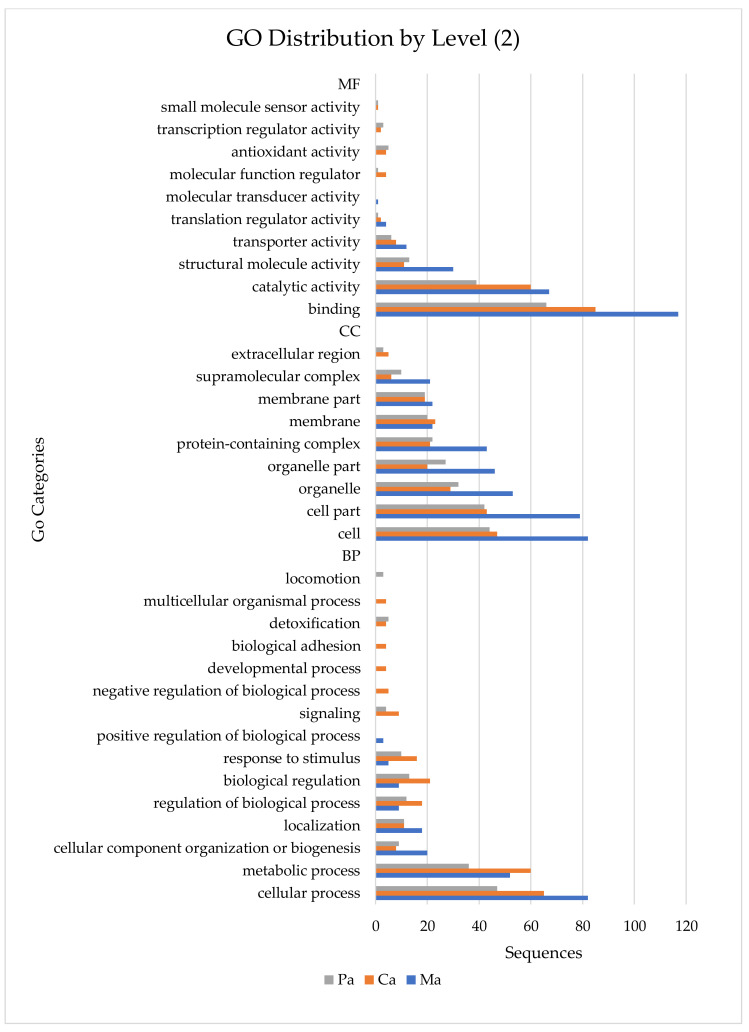
Gene ontology (GO) annotation per Blast2GO software according to GO distribution by level 2. Number of sequences associated with the three GO categories (MF—Molecular Function, CC—Cellular Component, BP—Biological Process) in the three ascidians’ samples (Ca—*Ciona* sp., Ma—*Molgula* sp., Pa—*Microcosmus* sp.).

Proteins identified with Proteome Discoverer software were blasted and mapped using Blast2Go software. They were annotated into three main categories of GO distribution by level 2: Cellular Components (CC), Biological Process (BP), and Molecular Function (MF) (Figure 2). In a global analysis, 34 GO terms were detected. Overall, the most represented MF associated GO terms were binding, catalytic activity and structural molecule activity in all ascidians’ species. In CC category, cell, cell part and organelle Go terms were the most represented, in all samples. In BP category some differences between species were found, being cellular process, metabolic process, and cellular component organization or biogenesis the most represented GO terms in *Molgula* sp. whilst cellular process, metabolic process and biological regulation were the most represented ones in *Ciona* sp. and *Microcosmus* sp. (Figure 2). Detailed information of GO analyses can be found in Supplementary Table S1.

### 2.3. Kyoto Encyclopedia of Genes and Genomes Analyses

Kyoto Encyclopedia of Genes and Genomes (KEGG) analyses revealed 29 different enzymes involved in 38 different pathways. The highest number of KEGG pathways, 38, were found in *Ciona* sp. while in *Molgula* sp. and *Microcosmus* sp. samples, 17 and 13 pathways were identified, respectively (Table 2). The highest number of enzymes detected were associated to the biosynthesis of antibiotics, purine metabolism and Glycolysis/Gluconeogenesis pathways in the same order. There were 10 KEGG pathways present in all ascidian species: biosynthesis of antibiotics, purine metabolism, glycolysis/gluconeogenesis, thiamine metabolism, methane metabolism, pentose phosphate pathway, glyoxylate and dicarboxylate metabolism, Programmed cell death 1 (PD1) and its ligand(PD-L1) checkpoint pathways in cancer, T cell receptor signaling pathway, and T helper type 1 (Th1) and type 2 (Th2) cell differentiation (Table 2).

Carbon fixation in photosynthetic organisms, Citrate cycle (TCA cycle), Drug metabolism—other enzymes, Fructose and mannose metabolism, one carbon pool by folate, Pyrimidine metabolism and pyruvate metabolism pathways were only found in *Ciona* sp. and *Molgula* sp. samples (Table 2 and Supplementary Table S2). Glutathione metabolism, Phenylpropanoid biosynthesis, and Tryptophan metabolism pathways were only detected in *Ciona* sp. and *Microcosmus* sp. samples (Table 2 and Supplementary Table S2). There were no KEGG pathways only detected in *Molgula* sp. and *Microcosmus* sp. samples.

### 2.4. Associated Organisms from a Metaproteomic Perspective

**Figure 3:**
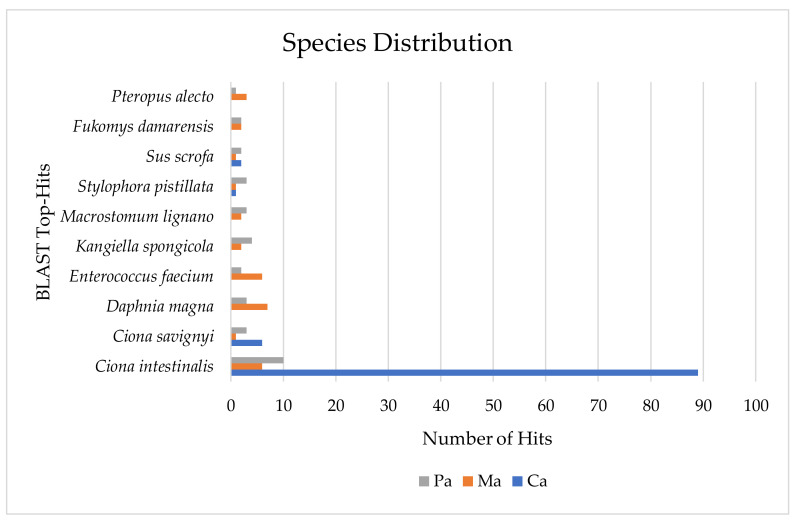
Top 10 hits of species protein distribution found in the three studied ascidians (Pa—*Microcosmus* sp., Ma—*Molgula* sp., and Ca—*Ciona* sp.) identified through the species distribution tool from the Blast2GO software.

Among the ascidians’ specimens studied, several associations with microorganisms are described. In this respect, a metaproteomic analyses of the identified proteins was performed using the species distribution tool available within Blast2GO software. All proteins identified were associated to a total of 222 unique taxonomic sources through the description of their respective BLAST-hits. The most represented taxonomic level corresponded to *Ciona intestinalis* (105 proteins) followed by *Ciona savignyi* and *Daphnia magna*, both with 10 proteins, while 8 and 6 proteins were ascribed to the *Enterococcus faecium* and *Kangiella spongicola*, respectively (Figure 3).

**Figure 4:**
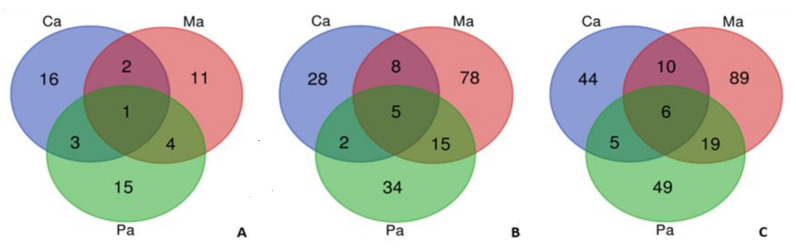
Species distribution analyses obtained with Blast2GO software showing unique and shared taxonomic origins of the identified proteins from three ascidian species (Ca—*Ciona* sp., Ma—*Molgula* sp., and Pa—*Microcosmus* sp.). Venn diagrams built with taxonomic assigned proteins to (**A**) Bacteria, (**B**) Metazoan, and (**C**) total number of taxonomic levels from hits description.

From those, 52 hits belong to Bacteria (1 uncultured bacterium and 1 *Candidatus* Rokubacteria bacterium) while 170 are Eukaryota hits. A total of 40 hits (30 Eukaryota and 10 Bacteria hits) were shared between ascidians’ samples while 182 hits only appeared (one or more sequences) in just one sample (140 Eukaryota and 42 Bacteria hits) (Figure 4). Through the mentioned species distribution tool, 124 different taxa were observed in *Molgula* sp., 79 and 65 different taxa in *Microcosmus* sp. and *Ciona* sp., respectively. Regarding the total number of identified hits, *Ciona* sp. was the sample with the highest percentage of Bacteria hits detected (33.8%) while *Molgula* sp. had the highest percentage of Eukaryota hits found (85.5%). In an overall analysis, 17 different phyla were detected, 6 of those are Bacterial phyla while 11 phyla belong to Eukaryota domain. Only 7 phyla were present in all the three species, 5 Eukaryota and 2 Bacteria phyla (Table 3). The highest number of hits in all ascidians’ samples corresponds to Chordata phylum (Table 3). In total, Arthropoda was the second highest detected phylum. However, looking in particular to each sample, Proteobacteria had more hits than Arthropoda in two samples (*Ciona* sp. and *Microcosmus* sp.).

The same occurs with Cnidaria regarding Firmicutes phylum (Table 3). Bacteroidetes, Brachiopoda, Nemertea, and Porifera hits were only detected in *Molgula* sp. (Supplementary Table S3). On the other hand, Chlamydiae and Planctomycetes phyla were only detected in *Microcosmus* sp. Actinobacteria and Tardigrada hits were detected in *Ciona* sp. and *Microcosmus* sp., while Echinodermata and Mollusca phyla were detected in *Molgula* sp. and *Microcosmus* sp. (Supplementary Table S3). Regarding Bacteria species hits detected, as above-mentioned, the principal phylum reported was Proteobacteria (34 hits) belonging mostly to Gammaproteobacteria and Alphaproteobacteria classes. On the other hand, Chordata was the most detected Metazoan phylum.

### 2.5. Antimicrobial Peptides Identified with MaxQuant

The analyses with Andromeda search engine embedded in MaxQuant freeware allowed the clusterization of the previously identified proteins (by Proteome Discoverer) with Antimicrobial Peptides (AMPs). In total, 37 AMPs were detected within 311 proteinGroups (Table S4). The original MaxQuant output file containing all the identified proteins clustered together with AMPs can be found in Supplementary Table S5. Results from the mapping of the proteinGroups found in the three ascidian samples against a non-redundant AMPs database are described in Table 4. 37 AMPs were found and detected in 12 databases. From those 37 peptides, 10 were found in the UniProtKB and 6 peptides were found in the Antimicrobial Peptide Database (APD). Three peptides were found in each one of the following databases: Antiviral Peptides (AVP), Collection of Anti-Microbial Peptides (CAMP), Anuran Defense Peptides (DADP), Dragon Antimicrobial Peptide (DAMPD), and in the Automated Discovery Tool for Gene-Coded Antimicrobial Peptides (AMPer).

In the Database of Antimicrobial Activity and Structure of Peptides (DBAASP) and in a bacteriocin database (BAGEL-Bagel_I, Bagel_III), 2 peptides were found. Finally, 1 peptide was found in the Antimicrobial Sequences Database (AMSDb), and in the Yet Another Database of Antimicrobial Peptides (YADAMP). The AMPs detected are mostly related with antibacterial defense. Additional information of the identified AMPs can be found on Supplementary Table S4.

## 3. Discussion

### 3.1. Proteins Identified by Shotgun Proteomics

A total of 337 metazoan and 106 bacterial unique proteins were identified in the three ascidians specimen’s tunic (Supplementary Dataset S1). In general, the total number of proteins identified in this study was relatively less than in other ascidian proteomes [33,35,37]. The homogenization method and sample preparation can be improved. Some difficulties experienced for protein extraction in the tunic, as well as, the relative abundance or diversity of proteins within this tissue may have influenced the number of proteins identified. Noteworthy, ascidians tunic is mainly composed of cellulose with free cells [12]. However, in the previous works, the proteomic studies were conducted on different samples: three embryonic stages (unfertilized eggs, 16 cell-stage embryos, and tadpole larvae) of *C. intestinalis* [33], inner bodies of *Microcosmus exasperatus* and *Polycarpa mytiligera* [37], and ovaries of *C. intestinalis* [35]. Nonetheless, despite being conducted on different samples, the number of proteins was similar as the case of *P. mytiligera* where 126 proteins where found regarding inner bodies [37]. In the present study, the best yield for protein identification corresponded to *C. intestinalis*. This species is considered as a model organism in developmental and evolutionary studies which may explain the highest number of proteins found compared to the other species studied (Figure 1, Figure 3, Supplementary Dataset S1). To date, in the National Center for Biotechnology Information (NCBI) database only 5 tunicate genomes are available, 4 of which belonging to ascidians (*Botryllus schlosseri*, *Phallusia mammillata*, *Ciona savigny,* and *Ciona intestinalis*) contrasting with the 14 annotated genomes currently available at Ascidian Network for In Situ Expression and Embryological Data (Aniseed) database [38]. In fact, the information of protein sequences that can be used as a complement database to study the tunic proteome is still scarce.

From the 443 unique proteins detected only 34 of them were shared, 25 metazoan and 9 bacterial proteins (Figure 1). Most of these shared proteins are ubiquitous such as actin, tubulin, histones, and ATPase (Table 1) that play fundamental roles in basal metabolism. Apart from those genes encoding proteins associated with housekeeping functions [28], unique proteins, both metazoan and bacterial, revealed some species-depending differences. *Ciona* sp. and *Microcosmus* sp. had the highest and the smallest number of proteins detected (Figure 1), as well as the number of KEEG pathways found (Table 2), respectively. Although the proteome is affected by the environmental conditions, it should be undervalued since two of those studied ascidians were collected in the same sampling point. Most of the differences found could be explained by the biology of the species, tunic features, and associated organisms. The diversity of ascidians microbiomes containing species specificity has been previously reported [39]. As happened in our study, Kuplik et al., (2019) also verified that different ascidians have different proteome profiles [37].

Overall, ascidians tunic seems to be mostly composed by structural proteins though revealed to be metabolically active with the presence of several enzymatic pathways within it, revealing the potential occurrence of a higher level of biological interactions and processes occurring in that tissue (Table 2). In fact, metabolic activity was one of the most represented GO terms in the BP category of Blast2GO analysis in all the studied specimens. Moreover, AMPs were also found, as well as serine proteases and serine proteases inhibitor among the shared proteins (Figure 1, Table 1). Besides, some proteins are uncharacterized, whose conserved domains are homologues to calcium binding proteins, DNA-binding, and RNA polymerase II C-terminal domain (CTD) heptapeptide repeat phosphatase activity (Table 1). Some of these proteins may play important roles in the interaction of host-associated microorganisms.

### 3.2. Distribution of Bacterial Proteins among Species

One of the main advantages of applying metaproteomics is to indirectly explore the presence of microorganisms through the taxonomic information of each protein, as well as infer the functional state of the microbial community [41]. All the proteins identified in this study were associated to taxonomic sources through the description of their respective BLAST-hits using the species distribution tool provided by the Blast2GO software (version 5.2.5).

Within *Ciona* sp. results, 65 of the 182 proteins were assigned to taxonomic hits. *Ciona* sp. showed to have the highest percentage of unique bacterial BLAST-hits of the species distribution, 22 in a total of 33 proteins. Specific KEGG pathways were detected in *Ciona* sp. tunic as the carbon fixation in photosynthetic organisms and carbon fixation pathways in prokaryotes. These findings might be associated to organisms within *Ciona* sp. tunic involved in photosynthetic processes. On the other hand, *Molgula* sp. samples had 18 of the 44 reported bacterial hits assigned to a taxonomic description. KEGG pathways, in special, Carbon fixation in photosynthetic organisms was one of the pathways with high number of enzymes, as described above. *Microcosmus* had 23 out of the 39 bacteria proteins with a taxonomic description, being the sample with the highest diversity of bacterial phyla detected. The number of KEGG pathways, 38, may be correlated with such diversity within this tunic tissue. Focusing on the diversity of the detected phyla, in the three samples, Proteobacteria was the most detected bacterial phylum, with a highlight to Alphaproteobacteria and Gammaproteobacteria classes (Table 3, Table S3). The results here obtained regarding species distribution are in accordance with ascidians microbiome studies that have been published [13,14,17]. In those studies, whose aim is the analysis of the bacterial community present in ascidians tunic, Proteobacteria is regularly one of the most detected phyla. The other bacterial phyla detected in the present study (Firmicutes, Chlamydiae, Actinobacteria, Bacteroidetes, and Planctomycetes) have also been attributed to ascidians microbiome community (Table 3, Table S3). Biosynthesis of antibiotics was one of the most detected KEGG pathways. Ascidians are well known for being associated with the production of bioactive compounds. These metabolites have now been related with the associated ascidian organisms and several important functions, among them the contribution to host defense against potential pathogens.

### 3.3. Interaction Host–Microbes

Tunicates, as happens with all invertebrates, do not have an adaptive immune system depending only on the innate immunity, which consists in cellular and humoral components [42,43]. Therefore, tunic represents a natural protective barrier and the first line of response to any injury, microbial association, or environmental change. This absence of an adaptive immune system might justify the presence of proteins associated to innate immunity detected in the samples, suggesting that host defense mechanisms were activated at the sampling moment.

The metazoan Down syndrome cell adhesion molecule-like protein was detected in *Molgula* sp. samples. This protein has been associated with immune mechanisms of invertebrates mediating phagocytosis and adherence of bacteria [44,45]. Proteins suggested to be involved in humoral immune responses were also present in *Ciona* tunic proteome. Among those proteins are “Hemocyanin-like protein 2” and “Barrier to autointegration factor” (BAF), both grouped in the MF category. The involvement of BAF in innate immune response as an inhibitor of exogenous viral DNA replication has been described; thus despite having an important role during cell cycle is also involved in host defense response [46]. Hemocyanin-like protein 2 (Fragment) is also grouped in Catalytic activity GO term of MF category and in Metabolic Process GO term in BP category present in *Ciona* proteome. Some authors suggest “Hemocyanin-like protein 2” to function as phenoloxidase [47] which is synthesized as prophenoloxidase, it has been also proposed that its activation leads to humoral immune response and melanogenetic pathway in invertebrates [48,49]. Moreover, variable region-containing chitin-binding protein (VCBPB2) was detected in *Ciona* tunic proteome and previous studies have detected VCBP expression in epithelial cells from stomach, intestine, and associated with the immune system [50]. VCBP is supposed to be enrolled in the recognition of pathogens and anticipatory immunity [44].

On the other hand, “mannan-binding lectin serine protease 1-like” protein could be detected, in the present study, in *Ciona* sp. sample tunic. In literature, it has been described that this protein has a serine-type endopeptidase activity and it is associated to the lectin pathway enrolled in innate immunity and host defense [51,52,53,54]. Interestingly, it is present in the same tissue, proteins with the opposite activity. In *Ciona* sp. samples, proteins with serine-type endopeptidase inhibitor activity were also detected as “A disintegrin and metalloproteinase with thrombospondin motifs adt-1”, Aprotinin, “Inter-alpha-trypsin inhibitor heavy chain H3”, “Complement component C3”, and “Alpha-2-macroglobulin homologue”. These proteins were associated to biological regulation GO term in BP category.

However, due to redundancy, several of those proteins with endopeptidase inhibitor activity were associated to negative regulation of biological process group but also aprotinin and “IF rod domain-containing protein” associated with “negative regulation of inflammatory process” and “complement activation, lectin pathway” GO processes were present in this group. Detoxification GO term detected in *Ciona* sp. encompasses essentially proteins with peroxidase and peroxiredoxin activity, in *Microcosmus* sp. prevailed catalase proteins in this group. Another interesting protein found in *Ciona* sp. samples was spermidine/putrescine ABC transporter substrate-binding protein associated to a Gram-negative bacterium, *Pelagicola* sp. strain LXJ1103. The presence of polyamine uptake systems as ABC may suggest also the presence of polyamines as the case of spermidine and putrescine which have been revealed to be important in pathogenesis processes [55]. Spermidine and putrescine are polyamines described to be enrolled in prokaryotic and eukaryotic cell growth as it is well documented in *Escherichia coli* model, polyamines have been described to be essential cellular components [56,57].

In *Microcosmus* sp. samples, Ca^2+^ binding repeats-in-toxin (RTX)toxin-like protein was detected. This protein revealed high similarity with *Rhizobium subbaraonis* being documented as a virulent factor with cytotoxic and hemolytic activity and produced by a huge portion of Gram-negative bacteria [58,59,60]. This protein has also being associated to pathogenesis; a current hypothesis is that it is correlated with calcium levels dysregulation leading to cytoskeletal destruction and subsequently to cell killing [61]. Contrasting with the presence in *Microcosmus* sp. sample of an aprotinin protein with serine-type endopeptidase inhibitor activity detected in Metazoa section, a lysyl endopeptidase with high similarity with *Pseudomonas aeruginosa* was detected in Bacteria section in *Microcosmus* sp. samples. The presence of proteins with antagonistic effect suggests the occurrence of regulatory reactions promoting homeostasis [62].

As above-mentioned, biotechnological potential has been associated with marine invertebrate organisms, and ascidians are not an exception. The production of AMPs is suggested to be related with innate immunity acting as a chemical barrier against pathogens [42]. A crucial question is to know who the true producer is, the host or the associated microorganisms. With the current study, we suggest the occurrence of reaction defenses of all studied hosts and bacteria present in their tunic against other associated organisms in the studied tunic samples. Those analyses showed a huge potential for AMPs to be produced as a response to bacterial invasion or bacteria combating against other bacteria/microorganisms. Indeed, in the studied samples, the presence of AMPs either produced by metazoan or bacterial organisms was detected. In the present study, the most representative AMPs were i) C-type lysozyme/alpha-lactalbulmin family, ii) core histones H2B, iii) antibacterial peptides, and iv) cationic AMPs (Cathelicidin type). Most of the detected AMPs are produced by eukaryotic organisms, however, bacteriocins which have a bacterial origin were also detected. The identified bacteriocins were Lichenicidin (lantibiotic-type) and Bacteriocinalbusin (lectin-type). Bacteriocins consist in antibacterial peptides ribosomally encoded, usually targeting close phylogenetic relatives [63,64]. These molecules have gained a renewed attention as potential antibiotics to overcome several multiresistance problems happening with the available ones [64,65,66]. Lichenicidin bacteriocin has already demonstrated activity against Methicillin resistant *Staphylococcus aureus* strains and other Gram-positive bacteria [67,68]. Lectin-type bacteriocins are usually secreted by Proteobacteria [66]. The presence of bacterial origin-based AMPs leads us to suggest that bacteriocins, in concrete, are probably produced by bacteria present in the ascidians tunic to inhibit or control the development and growth of other non-desirable bacteria or pathogens, contributing to the host defense mechanism. It is possible that the applied proteomic technique, failed to detect non-ribosomal peptides which frequently have been associated to bioactive activity. Other fact that supports the biotechnological potential activity occurring in all the studied ascidians tunic is that the principal KEEG pathway had been the “biosynthesis of antibiotics”; the pathway with the highest number of enzymes detected in all species.

In the present study, it was possible to characterize the proteome of three different ascidians specimens. This exploratory study revealed a high potential for high-throughput characterization and biodiscovery of ascidians’ tunic and its microbiome. Through the study of the proteins present in a sample it is possible to analyse the immediate effects caused by environmental changes or other organisms. There is still a lack of proteomic approaches in the microorganism association studies, however the applied method encompassing both bacterial and metazoan proteins led to infer and understand the occurrence of host–microbe interactions. The highest diversity of proteins and pathways found are supported by the diversity of the detected phyla. The present study shows the complex and diverse interactions occurring in three ascidians tunics. We suggest that besides the possible different environments from which each ascidian had been collected may contribute to such diverse proteomes found, specific microbial associations and the innate immunity system of each ascidian have also a huge impact on the establishment of those associations with biotechnological and pharmaceutical importance.

## 4. Materials and Methods

### 4.1. Sampling and Protein Extraction

Three ascidians species were collected at Porto de Leixões (*Ciona* sp. and *Molgula* sp.) and Peniche (*Microcosmus* sp.), Portugal. Specimens were brought to the laboratory inside zip bags in fresh conditions to be dissected. Three small pieces from different parts of each tunic specimen were cut and pooled. Then, 0.5 g from the tunic of each species were added into lysis microtubes (Lysis Tube with impact beads, Analytik Jena AG, Jena, Germany), containing the digestion buffer. Sodium dithionite (SDT) buffer (2% Sodium dodecyl sulfate (SDS), 100 mM Tris(hydroxymethyl)aminomethane hydrochloride (Tris/HCl) pH 7.6, 0.1 M Dithiothreitol (DTT)) and protease inhibitors (PIs, Roche, 11697498001). Tunic tissues were disrupted and homogenized in a cold support using the SpeedMill PLUS homogenizer (Analytik Jena AG, Jena, Germany) in continuous mode (3 cycles, 1 min each) and incubated overnight at room temperature. Afterwards, samples were vortexed; heated for 3 min at 95 °C and subsequently centrifuged at 16000 g, for 20 min. Finally, the supernatant was collected, and the total protein concentration was estimated according to the Bradford method [69]. Extracted proteins were stored at –20 °C. The extracted proteins, of the three ascidian species, were processed in duplicates on the Filter Aided Sample Preparation (FASP) protocol described by Wisniewski et al. (2009) (15). In resume, this approach comprised the alkylation and digestion of 30 µg of the extracted proteins with trypsin (recombinant, proteomics grade, Roche, Basel, Switzerland) at an enzyme to protein ratio of 1:100 (*w/w*) for 16 h at 37 °C using centrifugal filter units with nominal molecular weight limit (NMWL) of 10 kDa (MRCPRT010, Millipore, Billerica, MA, USA). Through centrifugal filtration, peptides were recovered and acidified with Trifluoracetic acid (TFA: 10% *v/v*). Samples were desalted and concentrated by reversed-phase extractions (C18 Tips, 100 µL, Thermo Scientific, 87784, Bremen, Germany) with acetonitrile (ACN: 50% *v/v*) and TFA (0.1% *v/v*) for peptide elution. Before LC-MS/MS analysis, samples were dried in the speed-vac and resuspended in formic acid (FA: 0.1% *v/v*) to a final concentration of 0.04–0.06 μg/μL.

### 4.2. LC-MS/MS Analyses

FASP protein digests were processed using a nano LC-MS/MS, composed by an Ultimate 3000 liquid chromatography system coupled to a Q-Exactive Hybrid Quadrupole-Orbitrap mass spectrometer (Thermo Scientific, Bremen, Germany). Samples were loaded onto a trapping cartridge (Acclaim PepMap C18 100 Å, 5 mm × 300 µm i.d., 160454, Thermo Scientific) in a mobile phase of 2% ACN, 0.1% FA at 10 µL/min. After 3 min loading, the trap column was switched in-line to a 15 cm by 75 μm inner diameter EASY-Spray column (ES800, PepMap RSLC, C18, 3 μm, Thermo Scientific, Bremen, Germany) at 300 nL/min. Separation was generated by mixing A: 0.1% FA and B: 80% ACN, with the following gradient: 5 min (2.5% B to 10% B), 60 min (10% B to 35% B), 5 min (35% B to 99% B) and 5 min (hold 99% B). Subsequently, the column was equilibrated with 2.5% B for 12 min. Data acquisition was controlled by Xcalibur 4.0 and Tune 2.8 software (Thermo Scientific, Bremen, Germany). The mass spectrometer was operated in data-dependent (dd) positive acquisition mode alternating between a full scan (*m/z* 380–1580) and subsequent HCD MS/MS of the 10 most intense peaks from full scan (normalized collision energy of 27%). Electrospray ionization (ESI) spray voltage was 1.9 kV and capillary temperature was 275 °C. Global settings: use lock masses best (*m/z* 445.12003), lock mass injection Full MS, chrom. peak width (Full width at half maximum—FWHM) 15s. Full scan settings: 70k resolution (*m/z* 200), automatic gain control (AGC) target 3e6, maximum injection time 50 ms. dd settings: minimum AGC target 8e3, intensity threshold 7.3e4, charge exclusion: unassigned, 1, 8, >8, peptide match preferred, exclude isotopes on, dynamic exclusion 20 s. MS2 settings: microscans 1, resolution 35k (*m/z* 200), AGC target 2e5, maximum injection time 110 ms, isolation window 2.0 *m/z*, isolation offset 0.0 *m/z*, spectrum data type profile (Figure S1).

### 4.3. Protein Identification

Raw data (6 Orbitrap) corresponding to the two technical replicates of the three ascidians species were analysed and processed using Proteome Discoverer 2.2.0.388 software (Thermo Scientific) and searched against the UniProt Knowledgebase (UniProtKB) for the Metazoa and Bacteria taxonomic selection (2018_07 release). The Sequest HT search engine was used for protein identification. The ion mass tolerance was 10 ppm for precursor ions and 0.02 Da for-fragment ions. Maximum allowed missing cleavage sites was set to 2. Cysteine carbamidomethylation was defined as constant modification. Methionine oxidation and protein N-terminus acetylation were defined as variable modifications. Peptide confidence was set to high. The processing node Percolator was enabled with the following settings: maximum delta Cn 0.05; decoy database search target FDR 1%, validation was based on q-value. The identification of the shared proteins among the analysed replicates was achieved using an online free tool to construct Venn diagrams, available at the webserver of the Bioinformatics and Evolutionary Genomics Center (BEG/Van de Peer Lab site), Ghent University, Belgium, http://bioinformatics.psb.ugent.be/webtools/Venn/.

### 4.4. MaxQuant Analyses

The same raw data (6 Orbitrap) were also mapped with Andromeda search engine embedded in MaxQuant freeware (version 1.6.2.3) against an Antimicrobial Peptides (AMPs) database together with a custom database built with the proteins identified previously with the Proteome Discoverer software. The AMPs database groups 16,990 AMPs sequences that were carefully gathered from 25 AMP databases by Aguilera-Mendoza et al. [40]. Proteins identification was achieved applying the following parameters on MaxQuant freeware software (version 1.6.2.3): MS and MS/MS tolerances of 20 ppm and 0.5 Da, respectively; two missed tryptic cleavages were allowed; PSMs were accepted at a 1% false discovery rate (FDR) and trypsin was selected for protein cleavage. Carbamidomethylation was selected as static modification, while Oxidation of Methionine and Acetylation of protein N-terminus were chosen as variable modifications. The Posterior Error Probability (PEP) of proteinGroups was calculated using the script maxquant_pepcalc, available at https://github.com/pstew/maxquant_pepcalc.

### 4.5. Gene Ontology and Kyoto Encyclopedia of Genes and Genomes (KEGG) Analyses

The functional annotation of the identified proteins was achieved using the Blast2GO software (version 5.2.5, http://www.blast2go.com/) [70]. To group proteins according to biological process (BP), cellular component (CC), and molecular function (MF) domains, level 2 of Gene Ontology (GO) was applied. The involved enzymatic pathways were analyzed with the Kyoto Encyclopedia of Genes and Genomes (KEGG) analyses [71,72,73].

## 5. Conclusions

In the present work, a shotgun proteomics approach revealing the proteomic composition of three ascidians outer tunic was applied. This methodology showed to be suitable to characterize the whole proteome of the tunic giving insights into the interactions between hosts and their associated microorganisms. In total, 337 metazoan and 106 bacterial proteins were identified, as well as 37 AMPs. Most of the identified proteins, both from eukaryotic and prokaryotic origins, are mainly involved in basal metabolism. However, some identified peptides were related to AMPs from eukaryotic origin, with exception of bacteriocins. These AMPs could be produced by the tunic as a mechanism of self-protection against pathogens or to control associated organisms’ growth. The presence of bacteriocins can be associated with bacteria in the ascidian’s tunic, releasing them as potential antibiotics to inhibit the growth or colonization of other non-desirable bacteria or pathogens. The secretion of these AMPs and other antagonist proteins, like serine proteases and its inhibitors, could be part of the mechanism of microbial association contributing also to the host defense. The outcomes of this work revealed the tunic as a very active tissue in terms of bioactive compounds production. This approach can be useful to unravel the main metabolic pathways of the tunic and associated microorganisms, giving clues of microbiome composition and its potential interactions with the host. Although the present work constitutes an exploratory study, the approach employed revealed high potential for high-throughput characterization and biodiscovery of ascidians’ tunic and its microbiome. Hence, the outcomes of this work will certainly be useful to the scientific community for future studies involving a larger and representative sampling dataset.

## Figures and Tables

**Table 1:** Shared proteins among the three ascidian species (Ca—*Ciona* sp., Ma—*Molgula* sp., Pa—*Microcosmus* sp.) per Universal Protein Resource Knowledgebase (UniProtKB) taxonomic sections: Bacteria and Metazoa.

UniProtKB Taxonomic Section ^1^	Species ^2^	Accession Number ^3^	Protein Description ^4^
**Bacteria**	Ca, Ma, Pa	A0A1E2TY30	Actin, cytoplasmic 2
	Ca, Ma	A0A2I6SAN9 A0A2D9B6R8 A0A368ML82	Ubiquitin Histone domain-containing protein Histidine kinase
	Ca, Pa	A0A0A8RA46 A0A293NCX8	Lysyl endopeptidase, EC 3.4.21.50 TAF domain-containing protein
	Ma, Pa	A0A2V2P8C5 A0A318CZJ6 A0A1C7PFN9	Tubulin domain-containing protein Myosin_tail_1 domain-containing protein Tubulin_C domain-containing protein
**Metazoa**	Ca, Ma, Pa	A0A287B5W2 A0A1W5BGH3	Trypsinogen isoform X1 Myosin-10 isoform X2
	Ca, Ma	A0A182L962 A0A2U3WDE6 A0A368GTV8 F6PP44 F6KMG7 F7D7P2 A0A2Y9E533 L7N0S7	Uncharacterized protein Histone H2B Ribosomal protein S3 Fructose-bisphosphate aldolase, EC 4.1.2.13 Actin Uncharacterized protein Uncharacterized protein LOC101342124ATP synthase subunit alpha
	Ca, Pa	F6SM47 B0LXF7 A0A1W2W3E0 A0A1W5BMF0 A2SY09 A0A1W3JRI0 A0A1W3JCQ2 A0A1W3JCW3	Dolichyl-diphosphooligosaccharide--protein glycosyltransferase 48 kDa subunit, Oligosaccharyl transferase 48 kDa subunit Aprotinin Tropomyosin, smooth muscle/fibroblast CTM1-like isoform X1 (tropomyosin, smooth muscle/fibroblast CTM1-like isoform X2) Talin-2 isoform X1 Actin (act protein isoform X1) r27a protein isoform X1 Glial fibrillary acidic protein isoform X1P-selectin-like
	Ma, Pa	L5K7V0 N6TCR8 A0A2U3Z9L8 Q95PQ7 A0A091CUF9 A0A212D7H3 A0A074ZCV8	Tubulin beta-2C chain Uncharacterized protein LOW QUALITY PROTEIN: actin, gamma 1 Intermediate filament protein C Keratin, type II cytoskeletal 1 MYH1 Uncharacterized protein

^1^ Taxonomic subsection of the UniProtKB where proteins were retrieved; ^2^ Ascidian species where proteins were found: Ca—*Ciona* sp., Ma—*Molgula* sp., Pa—*Microcosmus* sp; ^3^ Accession numbers of the identified proteins; ^4^ Brief description of the identified proteins.

**Table 2:** Top 15 Kyoto Encyclopedia of Genes and Genomes (KEGG) found in the three ascidians’ species: Ca—*Ciona* sp., Ma—*Molgula* sp., Pa—*Microcosmus* sp. N.F.—not found.

	Species	Ca		Ma		Pa	
Pathway		Enzymes in Pathway	Sequences of Enzymes	Enzymes in Pathway	Sequences of Enzymes	Enzymes in Pathway	Sequences of Enzymes
**Biosynthesis of antibiotics**		9	9	10	10	4	4
**Purine metabolism**		5	13	6	40	1	16
**Glycolysis/Gluconeogenesis**		4	4	6	6	1	1
**Carbon fixation in photosynthetic organisms**		3	3	4	4	N.F.	N.F.
**Methane metabolism**		3	3	2	2	1	1
**Pentose phosphate pathway**		2	2	1	1	2	2
**Glutathione metabolism**		2	3	N.F.	N.F.	2	3
**Thiamine metabolism**		1	8	1	33	1	16
**Drug metabolism-other enzymes**		2	2	1	1	N.F.	N.F.
**Pyruvate metabolism**		2	2	1	1	N.F.	N.F.
**Glyoxylate and dicarboxylate metabolism**		1	1	1	1	1	1
**PD-L1 expression and PD-1 checkpoint pathway in cancer**		1	1	1	1	1	1
**T cell receptor signaling pathway**		1	1	1	1	1	1
**Th1 and Th2 cell differentiation**		1	1	1	1	1	1
**Fructose and mannose metabolism**		2	2	1	1	N.F.	N.F.

**Table 3:** Description of the shared phyla with the respective number of detected hits in all the three ascidian samples: Ca—*Ciona* sp., Ma—*Molgula* sp., and Pa—*Microcosmus* sp.

	Species	Ca	Ma	Pa
Phylum				
**Chordata**		112	56	34
**Arthropoda**		10	41	18
**Proteobacteria**		15	14	19
**Nematoda**		9	12	5
**Platyhelminthes**		2	9	7
**Firmicutes**		1	7	3
**Cnidaria**		4	1	4

**Table 4:** Antimicrobial peptides detected in the three studied ascidians’ samples.

AMP_ID ^1^	PGs ID ^2^ (peptides)	PEP ^3^	Original Database ^4^	AMP Description/Main Activity ^5^
Overall_15444|DAMPD_732|11\DAMPD\DAMPD_548|H2B_LITVA	0 (4)	6.959e-119	DAMPD	**Core histone H2A/H2B/H3/H4** Defence response to bacteria G+/–
Overall_11263|CAMP_Validated_1724|CAMPSQ4167|Histone H2B			CAMP	**Core histone H2B** Defence response to bacteria G+/–
Overall_15445|DAMPD_733|11\DAMPD\DAMPD_549|H2B_RHASC			DAMPD	**Core histone H2B** Response to bacteria G+/–
Overall_15492|DAMPD_780|11\DAMPD\DAMPD_591|ANN2_AREMA	1 (1)	0.006	DAMPD	**Arenicin-2 from Arenicola marina (Lugworm)** Antibacterial G+/–
Overall_33701|Yadamp_1162|2043|Beta-defensin 20	2 (1)	2.752e-05	YADAMP	**Beta defensin** Antibacterial and Antiviral
Overall_31051|UniProtKb_1300|Q30KP3;Q8C5A7|DFB20_MOUSE			UniProtKB	**Beta defensin** Antibacterial
Overall_6716|Bagel_I_54|54.1|LichenicidinVK21A1	3 (1)	0.028	Bagel_I	**Lantibiotic alpha** Antibacterial
Overall_7055|Bagel_III_7|7.3|Bacteriocinalbusin_B	4 (1)	0.038	Bagel_III	**Bacteriocin** Antibacterial G+
Overall_32056|UniProtKb_2305|A1A547;E9QMH8;Q6R1Z2|PGRP3_MOUSE	188 (1)	0.038	UniProtKB	**Peptidoglycan recognition protein 3** (N-acetylmuramoyl-l-alanine amidase) Antibacterial G+
Overall_4110|APD_2229|AP00908|Dermatoxin DA1	194 (1)	0.037	APD	**Antibacterial peptide** Bactericidal towards mollicutes (wall-less eubacteria) and Gram-positive eubacteria
Overall_4109|APD_2228|AP00907|Dermatoxin A1			APD	**Antibacterial peptide** Bactericidal towards mollicutes (wall-less eubacteria) and Gram-positive eubacteria
Overall_496|AMPer_496|DMS1_PACDA|Dermaseptin PD-1-5 precursor			AMPer	**Frog skin active peptide** AMPs with a large spectrum of activities
Overall_495|AMPer_495|DMS1_AGAAN|Dermaseptin AA-1-1 precursor			AMPer	**Frog skin active peptide** AMPs with a large spectrum of activities
Overall_2680|APD_799|AP01717|Esculentin-2PRa	195 (1)	0.011	APD	**Antibacterial peptide** Antibacterial
Overall_3280|APD_1399|AP02257|Lysozyme	196 (1)	4.469e-05	APD	**C-type lysozyme/alpha-lactalbumin family (Kinocidin)** Antibacterial G+/-, Antifungal, Antiparasitic, Chemotactic
Overall_31618|UniProtKb_1867|P79294|LYSC_SAISC			UniProtKB	**C-type lysozyme/alpha-lactalbumin family** Bacteriolytic function (defence response to bacterium)
Overall_31613|UniProtKb_1862|P79239|LYSC_PONPY			UniProtKB	**C-type lysozyme/alpha-lactalbumin family** Bacteriolytic function (defence response to bacterium)
Overall_31593|UniProtKb_1842|P79180|LYSC_HYLLA			UniProtKB	**C-type lysozyme/alpha-lactalbumin family** Bacteriolytic function (defence response to bacterium)
Overall_31590|UniProtKb_1839|P79179|LYSC_GORGO			UniProtKB	**C-type lysozyme/alpha-lactalbumin family** Bacteriolytic function (defence response to bacterium)
Overall_31592|UniProtKb_1841|			UniProtKB	**C-type lysozyme/alpha-lactalbumin family** Bacteriolytic function (defence response to bacterium)
Overall_4269|APD_2388|AP02388|BPTI	197 (1)	4.185e-276	APD	**Bovine Pancreatic Trypsin Inhibitor** Antibacterial
Overall_1040|AMSDb_52|APRFR_BOVIN|APROTININ ANTIBACTERIAL FRAGMENTS			AMSDb	**Bovine Pancreatic Trypsin Inhibitor** Antibacterial
Overall_6268|AVPdb_1892|AVP1895|Coronaviridae	198 (1)	0.012	AVP	**Coronavirus S2 glycoprotein** Antiviral
Overall_6267|AVPdb_1891|AVP1894|Coronaviridae			AVP	**Coronavirus S2 glycoprotein** Antiviral
Overall_12077|CAMP_Validated_2538|CAMPSQ940|Defensin J1-1	206 (1)	5.523e-12	CAMP	**Gamma-thionin family: Defensin** Antibacterial and Antifungal
Overall_30115|UniProtKb_364|Q1KLX1|CAMP_PANTR	249 (2)	0.001	UniProtKB	**Cathelicidin AMP** Antibacterial G+/–
Overall_546|AMPer_546|FAL39_HUMAN|Antibacterial protein FALL-39 precursor (FALL-39 peptide antibiotic); (Cationic antimicrobial protein CAP-18) (hCAP-18) [Contains:;Antibacterial protein LL-37]			AMPer	**Cathelicidin Cathionic AMP** Antibacterial G+/–
Overall_17064|DB|DBAASP_1120|1242|Cathelicidin antimicrobial peptide			DBAASP	**Cathelicidin Cathionic AMP** Antibacterial
Overall_21373|DBAASP_5429|5781|cgUbiquitin	254 (2)	0	DBAASP	**Ubiquitin family** Immune response and inflammation and viral infection
Overall_3029|APD_1148|AP02030|cgUbiquitin			APD	**Ubiquitin family** Immune response and inflammation and viral infection
Overall_31667|UniProtKb_1916|O80288|LYS_BPPS3	290 (1)	2.899e-60	UniProtKB	**Lysozyme** Defence response to bacterium
Overall_31249|UniProtKb_1498|Q8CFB4;E9QJR4;Q8CFA4|GBP5_MOUSE	305 (1)	0.014	UniProtKB	**Lysozyme C** Bacteriolytic function (defence response to bacterium)
Overall_5857|AVPdb_1481|AVP1484|Coronaviridae	314 (1)	0.006	AVP	**Synthetic peptide targeting critical sites on the SARS-associated coronavirus spike protein responsible for viral infection** Antiviral
Overall_8456|CAMP_Patent_1315|Sequence 1188|US 6573361	317 (1)	0.038	CAMP	**Guanylate-binding protein** Response to bacterium and inflammatory process
Overall_14669|DADP_2528|SP_2629|Ranalexin-1Vb Ranalexin-Vb	318 (1)	0.001	DADP	**Frog antimicrobial peptide** Antibacterial
Overall_12734|DADP_593|SP_C0ILB1|Nigroain-I	319 (1)	0.001	DADP	**Frog antimicrobial peptide** Antibacterial
**Overall_**13517|DADP_1376|SP_E7EKC8|Hainanensin-1_2	320 (1)	0.004	DADP	**Frog antimicrobial peptide** Antibacterial

^1^ Accession of the Antimicrobial Peptide (AMP) identified by MaxQuant (Accession in the nr AMP database [40] | Accession in original database); ^2^ ProteinGroups identification to which each AMP belongs, and the corresponding number of peptides identified; ^3^ Posterior Error Probability; ^4^ Original databases from which the identified AMP was retrieved; ^5^ Brief description of the identified AMPs.

## Supplementary Materials

The following are available online at https://www.mdpi.com/1660-3397/18/7/362/s1, Figure S1: Proteome Discoverer 2.2.0.388 software (Thermo Scientific) output files. 12 output files comprising two replicates from the three studied species. 6 output files regarding Metazoa section and 6 associated to Bacteria section. Within each taxonomic section, each species has 2 output files. Files are named under the following designation “A_B” where A designates the species name (Ca—Ciona sp., Ma—Molgula sp. and Pa—Microcosmus sp.) and B to which section derives the file (Bacteria or Metazoa); Table S1: Detailed information of Gene ontology obtained with Blast2Go software. The present table includes the number and the respective accession number of the sequences associated to each GO term to each ascidian species (Ca–Ciona sp., Ma—Molgula sp. and Pa—Microcosmus sp.). The analysis is according to the three main categories of Go distribution by level 2: Cellular Components (CC), Biological Process (BP) and Molecular Function (MF); Table S2: Output files of Kyoto Encyclopedia of Genes and Genomes (KEGG) analyses. In the table is provided the number of enzymes (#Enzs in Pathway), the number of sequences of each enzyme (#Seqs of Enzyme), and their respective accession (Seqs) number associated to each pathway for each ascidian’ species (Ca—Ciona sp., Ma—Molgula sp. and Pa—Microcosmus sp.); Table S3: Output files from the species distribution tool available in Blast2Go software. It is presented the description of each species to their respective number of Blast hits (#BLAST Top-Hits) for each ascidian’ species (Ca—Ciona sp., Ma–Molgula sp. and Pa—Microcosmus sp.); Table S4: Detailed information of the Antimicrobial peptides’ analyses identified with MaxQuant. The table contains the complete description of the AMPs identified in the three studied species, their identification name in the databases (ID_Name), number of protein groups, original database from which were retrieved, their focus, main activity and peptide sequence; Table S5: The original MaxQuant output file containing all the identified proteins clustered together with AMPs.
