# Supplementary material for: Shotgun Proteomics of Ascidians Tunic Gives New Insights on Host–Microbe Interactions by Revealing Diverse Antimicrobial Peptides
Source: Mar Drugs. 2020 Jul 13;18(7):362. doi: 10.3390/md18070362 (PMC7401272; doi:10.3390/md18070362)
Supplement: Supplementary file 1 [file marinedrugs-18-00362-s001.zip › Supplementary Material.docx]

Supplementary Material: Shotgun proteomics of ascidians tunic gives new insights on host-microbe interactions by revealing diverse antimicrobial peptides

Ana Matos^1,2^, Dany Domínguez-Pérez^1^, Daniela Almeida^1^, Guillermin Agüero-Chapin^1,2^, Alexandre Campos^1,2^, Hugo Osório^3,4,5^, Vitor Vasconcelos^1,2^, and Agostinho Antunes^1,2,^*

^1^ CIIMAR/CIMAR, Interdisciplinary Centre of Marine and Environmental Research, University of Porto, Terminal de Cruzeiros do Porto de Leixões, Av. General Norton de Matos, s/n, 4450-208 Porto, Portugal; anabastosmatos@gmail.com (A.M.); dany.perez@ciimar.up.pt (D.D.-P.); danielaalmeida23@gmail.com (D.A.); gchapin@ciimar.up.pt (G.A.-Ch.); amoclclix@gmail.com (A.C.); vmvascon@fc.up.pt (V.V.); aantunes@ciimar.up.pt (A.A.).

^2^ Biology Department, Faculty of Sciences, University of Porto, Rua do Campo Alegre, s/n, 4169-007 Porto, Portugal.

^3^ Instituto de Investigação e Inovação em Saúde- i3S, Universidade do Porto, Rua Alfredo Allen, 208, 4200-135 Porto, Portugal; hosorio@ipatimup.pt (H.O.).

^4^ Ipatimup, Institute of Molecular Pathology and Immunology of the University of Porto, Rua Júlio Amaral de Carvalho, 45, 4200-135 Porto, Portugal.

^5^ Department of Pathology and Oncology, Faculty of Medicine, University of Porto, Al. Prof. Hernâni Monteiro, 4200-319 Porto, Portugal.

***** Correspondence: aantunes@ciimar.up.pt; Tel.: +351-22-340-1813

**Dataset S1**—Proteome Discoverer 2.2.0.388 software (Thermo Scientific) output files. 12 output files comprising two replicates from the three studied species. 6 output files regarding Metazoa section and 6 associated to Bacteria section. Within each taxonomic section, each species has 2 output files. Files are named under the following designation “A_B” where A designates the species name (Ca—*Ciona* sp., Ma—*Molgula* sp. and Pa—*Microcosmus* sp.) and B to which section derives the file (Bacteria or Metazoa).

**Table S1**—Detailed information of Gene ontology obtained with Blast2Go software. The present table includes the number and the respective accession number of the sequences associated to each GO term to each ascidian species (Ca—*Ciona* sp., Ma—*Molgula* sp. and Pa—*Microcosmus* sp.). The analysis is according to the three main categories of Go distribution by level 2: Cellular Components (CC), Biological Process (BP) and Molecular Function (MF).

**Table S2**—Output files of Kyoto Encyclopedia of Genes and Genomes (KEGG) analyses. In the table is provided the number of enzymes (#Enzs in Pathway), the number of sequences of each enzyme (#Seqs of Enzyme), and their respective accession (Seqs) number associated to each pathway for each ascidian’ species (Ca—*Ciona* sp., Ma—*Molgula* sp. and Pa—*Microcosmus* sp.).

**Table S3**—Output files from the species distribution tool available in Blast2Go software. It is presented the description of each species to their respective number of Blast hits (#BLAST Top-Hits) for each ascidian’ species (Ca—*Ciona* sp., Ma—*Molgula* sp. and Pa—*Microcosmus* sp.).

**Table S4**—Detailed information of the Antimicrobial peptides’ analyses identified with MaxQuant. The table contains the complete description of the AMPs identified in the three studied species, their identification name in the databases (ID_Name), number of protein groups, original database from which were retrieved, their focus, main activity and peptide sequence.

**Table S5**—The original MaxQuant output file containing all the identified proteins clustered together with AMPs.

**Figure S1**—Representative LC-MS chromatogram. The figure displays the Total Ion Current - TIC and the relative abundance of the sample peptide ions acquired during the chromatographic separation corresponding to: A and B—technical replicates of *Ciona* sp., C and D—technical replicates of *Molgula* sp., E and F—technical replicates of *Microcosmus* sp... The acquisition conditions are described at the main text in the materials and methods section.
