# Supplementary material for: Shotgun Proteomics of Ascidians Tunic Gives New Insights on Host–Microbe Interactions by Revealing Diverse Antimicrobial Peptides
Source: Mar Drugs. 2020 Jul 13;18(7):362. doi: 10.3390/md18070362 (PMC7401272; doi:10.3390/md18070362)
Supplement: Supplementary file 1 [file marinedrugs-18-00362-s001.zip › Supplementary Figures/Supplementary Figure S1.docx]

Figure S1 - Representative LC-MS chromatogram. The figure displays the Total Ion Current - TIC and the relative abundance of the sample peptide ions acquired during the chromatographic separation corresponding to: A and B – technical replicates of *Ciona* sp., C and D - technical replicates of *Molgula* sp., E and F - technical replicates of *Microcosmus* sp.. The acquisition conditions are described at the main text in the materials and methods section.

A

B

C

D

E

F
